# GCKR genetic variants and circulating FGF21 identify a metabolic risk signature in metabolic dysfunction-associated steatotic liver disease

**DOI:** 10.1186/s12876-026-04767-9

**Published:** 2026-04-13

**Authors:** Asmaa Mohamed Fteah, Doaa Mamdouh Aly, Mohamed A. Elrefaiy, Ali Abdel Rahim

**Affiliations:** 1 Clinical Chemistry Department, Theodor Bilharz Research Institute, Giza, Egypt; 2 Hepato-gastroenterology Department, Theodor Bilharz Research Institute, Giza, Egypt

**Keywords:** MASLD, MASH, GCKR, FGF21, genetic susceptibility

## Abstract

**Background & aims:**

Interindividual variability in metabolic dysfunction–associated steatotic liver disease (MASLD) onset and its progressive inflammatory stage, metabolic dysfunction–associated steatohepatitis (MASH), reflects a complex interplay between metabolic burden and inherited susceptibility. We investigated the combined impact of genetic variants in the glucokinase regulatory protein gene (GCKR) and fibroblast growth factor 21 (FGF21), together with circulating FGF21 concentrations, on susceptibility to MASLD and its progression to MASH.

**Methods:**

This case-control study enrolled 150 patients with MASLD, 150 patients with clinically suspected MASH, and 150 age- and sex-matched healthy controls. Genotyping of GCKR *rs1260326* and FGF21 *rs838133* polymorphisms was performed using DNA extracted from peripheral blood samples by real-time polymerase chain reaction, while serum FGF21 levels were quantified by enzyme-linked immunosorbent assay. Associations with metabolic characteristics, liver function indices, and fibrosis severity were examined using correlation analyses and multivariate logistic regression models.

**Results:**

The GCKR rs1260326 TT genotype and T allele carriers had an increased risk of MASH progression versus MASLD (AOR for TT genotype: 16.875; 95% CI: 6.339–44.921, *P* = 0.001), adjusted for age, sex, BMI, HOMA-IR and triglycerides level. Moreover, the TT genotype was associated with higher alanine aminotransferase levels and reduced markers of hepatic synthetic capacity. In parallel, carriers of AG genotype of FGF21 rs838133 exhibited a higher propensity for progression to MASLD (AOR: 18.622; 95% CI: 1.619-214.169, *P* = 0.019) and G allele accompanied by greater insulin resistance and unfavorable lipid profiles. Circulating FGF21 concentrations demonstrated a stepwise increase from controls to MASLD and MASH groups and showed good discriminatory performance in differentiating MASLD from controls (AUC = 0.821) and excellent accuracy in identifying MASH among healthy individuals (AUC = 0.929).

**Conclusions:**

Genetic variation in GCKR and FGF21, together with altered hepatokine signaling, contributes substantially to metabolic dysregulation and liver disease severity. Integrating genetic profiling with circulating biomarkers may offer a refined strategy for identifying individuals at high risk of MASLD progression and advancing precision-based approaches in metabolic liver disease.

## Introduction

Metabolic dysfunction-associated steatotic liver disease (MASLD), formerly known as nonalcoholic fatty liver disease (NAFLD), has emerged as one of the most prevalent chronic liver disorders worldwide. Its rapid rise parallels the global increase in obesity, insulin resistance, and type 2 diabetes, positioning MASLD as a major contributor to liver-related morbidity and mortality. Current estimates indicate that nearly one-third of the adult population is affected by hepatic steatosis, with a substantial proportion at risk of progressing to the inflammatory and fibrotic stage known as metabolic dysfunction–associated steatohepatitis (MASH), previously termed nonalcoholic steatohepatitis (NASH) [1]. This progressive form carries a markedly higher likelihood of cirrhosis, hepatocellular carcinoma, and liver-related death, underscoring the urgent need for improved strategies in risk stratification and early detection [2].

Despite its clinical relevance, MASLD is far from a homogeneous condition. While many individuals remain in a relatively benign steatotic stage, others experience rapid disease progression driven by intrinsic biological susceptibility and a convergence of metabolic stressors [3] including excessive intake of fructose and alcohol, which has been shown to aggravate high-fat diet-induced steatohepatitis and disrupt hepatic iron homeostasis [4]. Traditional clinical tools, including liver enzyme measurements and imaging, offer limited accuracy in distinguishing simple steatosis from active steatohepatitis. Although liver biopsy remains the diagnostic gold standard for MASH, its invasiveness and limited feasibility in large populations have fueled growing interest in noninvasive biomarkers and molecular determinants that better reflect disease activity and prognosis [5]. In this context, host genetic predisposition and hepatokine signaling have gained increasing recognition as key modulators of hepatic lipid handling and metabolic homeostasis and complementary contributors to disease heterogeneity [6]. Genome-wide association studies have consistently implicated variants within the glucokinase regulatory protein (GCKR) gene as influential determinants of hepatic glucose flux and de novo lipogenesis. The common *rs1260326* (P446L) variant reduces the inhibitory effect of GCKR on glucokinase, thereby enhancing glycolytic throughput and triglyceride synthesis within hepatocytes [7]. Although this variant is often associated with favorable glycemic traits, it paradoxically predisposes to hypertriglyceridemia and hepatic fat accumulation, suggesting a complex role in metabolic liver disease. Whether this genetic background also contributes to the transition from MASLD to MASH remains an important area of investigation [8].

Alongside genetic susceptibility, hepatokines have emerged as pivotal mediators linking hepatic metabolism with systemic energy balance. Fibroblast growth factor 21 (FGF21) is a liver-derived hormone that orchestrates adaptive responses to metabolic stress by promoting fatty acid oxidation, ketogenesis, and energy expenditure while restraining lipogenic pathways [9]. Circulating FGF21 levels are consistently elevated in obesity and fatty liver disease, a pattern widely interpreted as a compensatory response to metabolic overload. However, chronic elevation may also reflect a state of impaired tissue responsiveness, commonly described as FGF21 resistance [10]. Beyond its circulating concentrations, genetic variation within the FGF21 locus particularly the *rs838133* polymorphism has been associated with adverse metabolic traits and altered dietary preferences, raising the possibility that inherited differences in FGF21 signaling influence susceptibility to hepatic steatosis and disease progression [11].

Despite growing evidence implicating both genetic susceptibility and hepatokine dysregulation in MASLD, these factors are often investigated in isolation. GCKR polymorphisms influence hepatic glucose–lipid flux, while FGF21 reflects adaptive metabolic stress; however, their combined clinical relevance in MASLD remains poorly defined. The primary objective of this study was to identify the association between GCKR *rs1260326* and FGF21 *rs838133* polymorphisms and MASLD susceptibility compared to healthy controls as non-invasive genetic markers. Secondary objectives included evaluation of genetic association with disease progression from MASLD to MASH, analysis across different inheritance models, assessment of Hardy–Weinberg equilibrium, and multivariable-adjusted regression analyses controlling for metabolic confounders. To our knowledge, no prior study has evaluated rs1260326 (GCKR) and rs838133 (FGF21) simultaneously in a stratified MASLD–MASH study, the present work highlights their integrated contribution to disease-associated metabolic phenotypes in a real-world clinical study.

## Methods

### Study design and participants

A total of 473 individuals were screened for eligibility in this case-control study. After exclusion of 23 participants (not meeting inclusion/exclusion criteria or incomplete data), 450 subjects were included in the final analysis and categorized into three groups: 150 adult patients (≥ 18 years) with a confirmed diagnosis of MASLD, 150 patients with clinically suspected MASH. Patients presenting to the liver clinic were initially screened using abdominal ultrasonography for evidence of hepatic steatosis. The operational distinction between MASLD and clinically suspected MASH was established using non-invasive criteria aligned with the current guidance of the American Association for the Study of Liver Diseases [12], including:

- Imaging-confirmed steatosis; with pre-defined cut-off values used for steatosis grading and fibrosis staging, based on Controlled Attenuation Parameter (CAP) measurements obtained by transient elastography (FibroScan).
- Biochemical evidence of liver injury (elevated liver enzymes).
- Presence of metabolic risk factors, consistent with established diagnostic criteria.

And as a normal control group; 150 apparently healthy subjects were included in the study, who were free from history of fatty liver, diabetes and hypertension, with normal clinical and biochemical profiles.

Excluded patients were those testing positive for viral markers for hepatitis B and C, individuals with significant alcohol consumption (≥ 30 g/day in men and ≥ 20 g/day in women), those with recreational drug abuse, patients with confirmed autoimmune or cholestatic liver diseases, other secondary causes of steatosis, and those with history of past malignancies or recurrent/secondary tumors.

### Clinical and biochemical assessment

All participants underwent an initial screening visit that included medical history, physical examination and standardized anthropometric measures (height, weight, body mass index (BMI) and waist circumference), obesity was defined by the World Health Organization as BMI over 30 kg/m², with morbid obesity defined as a BMI of 40 kg/m² or higher [13]. Relevant comorbidities such as type 2 diabetes mellitus, arterial hypertension and dyslipidemia were carefully documented.

Routine laboratory analysis was performed for all participants; including liver and kidney function tests in the form of serum bilirubin level, alanine aminotransferase (ALT) activity, aspartate aminotransaminase (AST) activity, total protein, albumin, alkaline phosphatase (ALP), ᵞ-glutamyl transferase (GGT), urea and creatinine along with total cholesterol, high-density lipoprotein cholesterol (HDL‐C), low‐density lipoprotein cholesterol (LDL‐C) and triglycerides. All biochemical analyses were carried out on the same day using the Beckman Coulter AU 480 chemistry analyzer (Beckman Coulter Ireland Inc., Brea, CA, USA) in the clinical chemistry department of our facility.

Insulin resistance was assessed using the homeostatic model assessment of insulin resistance (HOMA-IR), calculated according to the formula: fasting plasma glucose (mmol/L) Ⅹ fasting serum insulin (mIU/L) / 22.5 [14]. Viral serology for hepatitis B and C viruses, as well as insulin level, was determined using a chemiluminescence immunoassay kit (Siemens Healthcare Diagnostics Inc., Tarrytown, NY, USA).

Complete blood count was performed using an automated hematology analyzer on Beckman Coulter AcT Diff cell counter (Beckman Coulter Ireland Inc., Brea, CA, USA).

### Genetic variants and biomarker analyses

All molecular analyses and allelic discrimination assays were conducted within the chemical pathology department, Cairo university hospitals, under controlled laboratory conditions adhering to standardized molecular diagnostics protocols to ensure data reproducibility and analytical precision. The genomic deoxyribonucleic acid (DNA) was meticulously extracted from peripheral blood samples using the GeneJET whole blood genomic DNA purification mini-kit (ThermoFisher Scientific Inc., CA, USA), strictly following the manufacturer’s standardized protocol. Genotyping of the candidate genetic variants was determined in all participants using predesigned assays with quantitative real-time polymerase chain reaction (qPCR) employing taqman genotyping assays (ThermoFisher Scientific Inc., CA, USA). Allelic discrimination of genetic variants of GCKR *rs1260326* (C > T, P446L) (Assay ID: C_2862880_1) and FGF21 *rs838133* (G > A) (Assay ID: C_8832415_10) was achieved using sequence-specific fluorescent taqman probes, according to the protocol proposed by [15] on the software of step one real-time PCR ABI-7500 instrument (Applied Biosystems, Foster City, CA, USA).

The DNA concentration and purity were determined using qubit fluorometric quantification assays (ThermoFisher Scientific Inc., CA, USA). To ensure consistency across all downstream genotyping assays, the DNA concentration was normalized to 20 ng/µL, and no samples were excluded due to poor DNA quality.

Strict quality control measures were applied throughout the process. Negative controls were included in parallel on each plate to preclude possible cross-contamination or genotyping bias. The overall genotyping call rate exceeded 95% for both SNPs. Samples that showed unsuccessful amplification or ambiguous genotype calls were reanalyzed and successfully resolved upon retesting, with no persistent genotyping failures.

### Evaluation of circulating FGF21 levels

Venous blood samples were obtained from all participants following an overnight fast under standardized conditions. Immediately after collection, samples were centrifuged at 2500 × g for 10 min at 4 °C and the resulting supernatant was carefully aliquoted into pre-labeled tubes and promptly stored at − 80 °C until subsequent biochemical assessment to maintain analyte integrity and prevent degradation. Serum FGF21 concentrations were determined using a commercially available human enzyme-linked immunosorbent assay (ELISA) kit validated for clinical research; (BT LAB, China) catalogue number: E1983Hu; with intra-assay CV < 8%, inter-assay CV < 10% and measuring range: 20–800 pg/mL. The serum FGF21 was detected following the manufacturer’s guidelines. No samples exhibited FGF21 concentrations below the assay’s lower limit of quantification.

### Statistical analysis

Data were coded and entered using the statistical package for the Social Sciences (SPSS) version 28 (IBM Corp., Armonk, NY, USA). Data was summarized using mean and standard deviation for normally distributed quantitative variables or median and interquartile range for non-normally distributed quantitative variables and frequencies (number of cases) and relative frequencies (percentages) for categorical variables. Comparisons between groups were done using analysis of variance (ANOVA) with multiple comparisons post-hoc test in normally distributed quantitative variables while non-parametric Kruskal-Wallis test and Mann-Whitney test were used for non-normally distributed quantitative variables [16]. For comparing categorical data, Chi square (χ2) test was performed. Exact test was used instead when the expected frequency is less than 5 [17]. Logistic regression analysis was performed to evaluate the association between the studied genetic variants and disease outcomes. Initially, crude odds ratios (ORs) with 95% confidence intervals (CIs) were calculated using univariate logistic regression. Subsequently, multivariate logistic regression models was constructed to adjust for predefined potential confounding variables. Covariates were selected based on their established association with metabolic dysfunction and disease progression, including age, sex, body mass index (BMI), diabetes status, HOMA-IR, and triglyceride levels. Adjusted odds ratios (AORs) with 95% CIs were then calculated and reported. Covariates were selected a priori based on biological plausibility and evidence from previous literature. Data was double checked for normality using normality plots and Shapiro Wilk test and Post-hoc Bonferroni test. Hardy–Weinberg equilibrium (HWE) was assessed in the control group using the chi-square test for both variants to evaluate genotype distribution consistency. A *p*-value more than 0.05 was considered indicative of equilibrium.

Correlations between quantitative variables were done using Spearman correlation coefficient [18]. ROC curve was constructed with area under curve analysis performed to detect best cutoff value of FGF-21 for detection of diseased liver. Logistic regression was done to detect independent predictors of diseased liver [19]. A *p*-value less than 0.05 was considered as statistically significant.

## Results

### Baseline characteristics of the study population

A total of 450 participants were included and categorized into three groups: healthy controls, patients with MASLD, and patients with clinically suspected MASH. No statistically significant differences were observed among the groups with respect to age or sex distribution, indicating appropriate matching. In contrast, marked differences were evident in anthropometric and metabolic parameters. Patients with MASLD and particularly those with clinically suspected MASH exhibited significantly higher body mass index, waist circumference, and prevalence of metabolic comorbidities, including type 2 diabetes mellitus and hypertension (*p* < 0.001 for all). Biochemical profiling further demonstrated a progressive deterioration in metabolic and hepatic indices across the disease spectrum. Compared with controls, both patients with MASLD and clinically suspected MASH showed significantly higher levels of fasting insulin, HbA1C %, HOMA-IR, aminotransferases, γ-glutamyl transferase, and adverse lipid parameters, with the most pronounced alterations ob1served in patients with clinically suspected MASH. These findings confirm the close association between metabolic dysfunction, hepatic injury, and disease severity (Table 1).

**Table 1:** Demographic data, clinical, laboratory and radiological data in the MASLD, MASH and normal controls

Covariate		Controls (*n* = 150)	MASLD (*n* = 150)	MASH (*n* = 150)	*P* value
Age (Years) *		46.51 ± 6.98	48.41 ± 10.43	48.25 ± 7.20	0.302
Sex	Male ^†^	30 (20%)	22 (14.7%)	36 (24%)	0.352
	Female ^†^	120 (80%)	128 (85.3%)	114 (76%)	
Diabetes (Yes/No)		0/150	24/126	24/126	< 0.001
Hypertension (Yes/No)		0/150	48/102	78/72	< 0.001
Anthropometric measurements					
Waist circumference (cm) *		86.15 ± 5.71	90.67 ± 6.45	96.28 ± 6.59	< 0.001
Length (cm) *		170.20 ± 3.64	170.56 ± 5.99	171.52 ± 5.16	0.253
Weight (kg) *		69.48 ± 3.36	87.80 ± 9.14	92.48 ± 7.93	< 0.001
BMI (kg/m²) *		23.99 ± 0.95	30.22 ± 3.30	31.44 ± 2.32	< 0.001
Laboratories					
Hemoglobin (g/dl) *		14.08 ± 0.91	11.61 ± 1.33	11.12 ± 1.47	< 0.001
TLC (×10⁹/L) *		6.48 ± 1.65	5.00 ± 1.39	5.20 ± 1.68	< 0.001
Platelet count (×10³/µL) *		281.01 ± 73.07	166.27 ± 18.05	183.17 ± 35.09	< 0.001
FBS (mg/dl) *		94.09 ± 7.05	88.68 ± 8.21	92.95 ± 34.49	0.249
HbA1C (%) *		4.85 ± 0.57	5.15 ± 0.47	5.62 ± 1.05	< 0.001
Insulin (µU/ml) *		10.21 ± 3.56	13.79 ± 3.50	24.57 ± 9.02	< 0.001
HOMA-IR *		2.33 ± 0.88	3.05 ± 0.93	6.33 ± 5.23	< 0.001
Total protein (g/dl) *		8.89 ± 0.31	8.71 ± 0.39	7.33 ± 0.55	< 0.001
Albumin (g/dl) *		4.57 ± 0.47	4.51 ± 0.30	4.27 ± 0.32	< 0.001
ALT (IU/L) *		22.47 ± 8.72	29.61 ± 8.59	48.03 ± 12.80	< 0.001
AST (IU/L) *		21.99 ± 6.19	28.87 ± 7.44	34.13 ± 12.02	< 0.001
Total Bilirubin (mg/dl) *		0.81 ± 0.18	1.00 ± 0.09	1.05 ± 0.10	< 0.001
Direct Bilirubin (mg/dl) *		0.16 ± 0.05	0.55 ± 0.13	0.52 ± 0.09	< 0.001
Uric acid (mg/dl) *		3.72 ± 0.59	4.97 ± 0.99	5.62 ± 1.25	< 0.001
GGT (IU/L) *		25.75 ± 7.00	41.84 ± 10.65	49.08 ± 17.66	< 0.001
Urea (mg/dl) *		23.65 ± 7.21	33.09 ± 7.77	40.67 ± 2.95	< 0.001
Creatinine (mg/dl) *		0.80 ± 0.13	0.99 ± 0.17	1.11 ± 0.12	< 0.001
Total cholesterol (mg/dl) *		151.61 ± 22.00	205.67 ± 37.58	197.53 ± 46.18	< 0.001
HDL-C (mg/dl) *		51.80 ± 4.79	42.24 ± 8.73	36.87 ± 10.31	< 0.001
LDL-C (mg/dl)		75.89 ± 21.23	109.05 ± 8.33	107.52 ± 17.29	< 0.001
Triglycerides (mg/dl) *		119.28 ± 25.93	157.47 ± 24.33	146.77 ± 24.58	< 0.001
Abdominal US Findings^†^					
Fatty liver		0 (0%)	92 (62.7%)	50 (33.3%)	< 0.001
Hepatomegaly		0 (0%)	46 (30.7%)	100 (66.7%)	< 0.001
Fibroscan Findings^†^					
Fibroscan S1		0 (0%)	42 (28.0%)	0 (0%)	< 0.001
Fibroscan S2		0 (0%)	94 (62.7%)	56 (37.3%)	
Fibroscan S3		0 (0%)	14 (9.3%)	94 (62.7%)	

^*^Data are represented as mean ± SD

^†^Data are represented as a number (Percent)

### Frequency distribution of GCKR *rs1260326* polymorphism and its clinical correlates

Our study revealed a significant difference in GCKR rs1260326 genotype and allele frequencies across the studied groups. The TT genotype and the T allele were markedly enriched in patients with clinically suspected MASH compared with both MASLD patients and healthy controls. Multivariate logistic regression adjusting for age, sex, BMI, HOMA-IR and triglycerides level showed TT genotype carriers had a 16.875-fold increased risk of MASH progression versus MASLD (95% CI: 6.339–44.921, *P* = 0.001), with T allele conferring 4.353-fold risk (95% CI: 2.735–6.927, *P* = 0.001). While The TT genotype and the T allele were significantly lower in MASLD patients compared with healthy controls. Post-adjustment, TT genotype and T allele carriers conferring a lower risk for MASLD versus healthy controls (95% CI: 0.000-0.258, *P* = 0.009), (95% CI: 0.041–0.455, *P* = 0.006) respectively (Table 2).

**Fig. 1:**
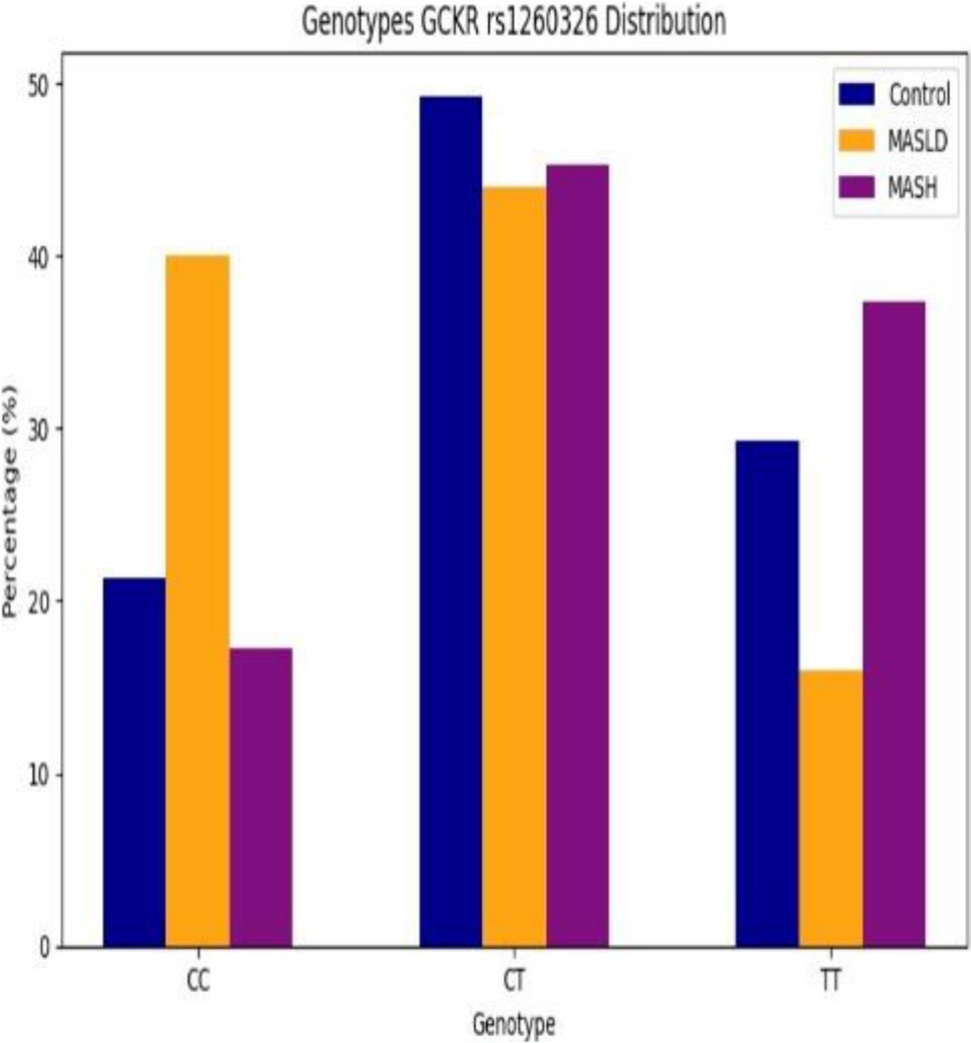
Genotypic distribution of *rs1260326* SNP variants among the studied groups

**Fig. 2:**
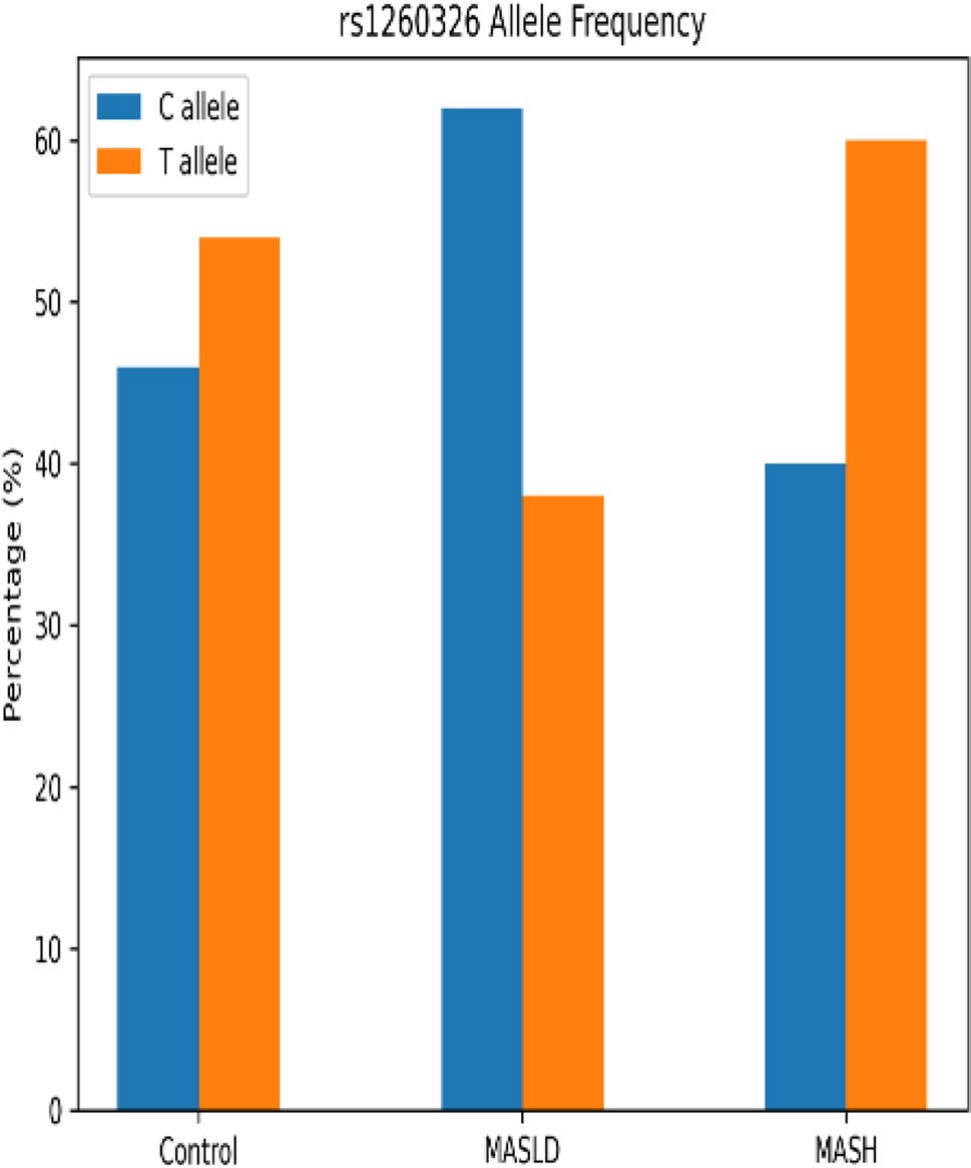
Allelic distribution of *rs1260326* SNP variants among the studied groups

**Fig. 3:**
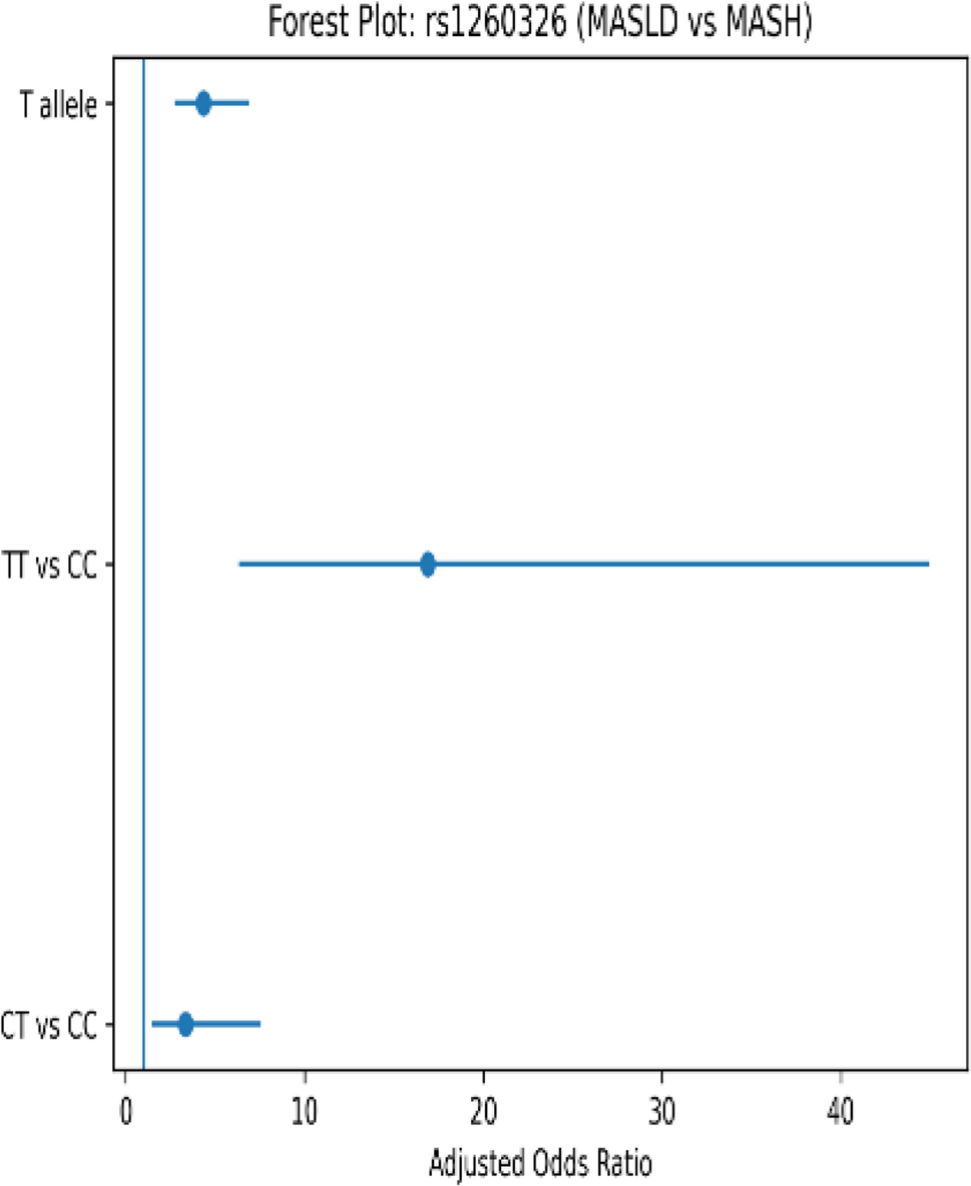
Forest plot of *rs1260326* SNP variants in MASLD versus MASH

These findings support a stage-dependent genetic effect of GCKR polymorphisms, influencing metabolic susceptibility differently from inflammatory disease progression. As well genotype frequencies in the control group were in Hardy–Weinberg equilibrium (*p* = 0.68) (Figs. 1, 2 and 3).

The TT genotype carriers exhibited higher mean ALT levels (*P* = 0.009) and lower total protein (*P* = 0.034) compared with CC and CT carriers. These findings suggest that the rs1260326 risk allele is not only associated with susceptibility to advanced disease but also correlates with markers of hepatocellular injury and reduced synthetic capacity (Table 3).

**Table 2:** Genotype and Allele distribution of *rs1260326* SNP genotypic variants and their odd’s ratios among the studied groups

Genotypes	Control (*n* = 150)		MASLD (*n* = 150)		MASH (*n* = 150)	*P* value
CC	32 (21.3%)		60 (40.0%)		26 (17.3%)	*0.005*
CT	74 (49.3%)		66 (44.0%)		68 (45.3%)	
TT	44 (29.3%)		24 (16.0%)		56 (37.3%)	

Alleles	Control (*n* = 300)		MASLD (*n* = 300)		MASH (*n* = 300)	*P* value
C	138 (46.0%)		186 (62.0%)		120 (40%)	*0.010*
T	162 (54.0%)		114 (38.0%)		180 (60%)	

Normal controls versus MASLD						
Codominant model	Control (*n* = 150)	MASLD (*n* = 150)	Crude OR (95% CI)	*P* value	Adjusted OR (95% CI)	*P* value
CC	32 (21.3%)	60 (40.0%)	Reference		Reference	
CT	74 (49.3%)	66 (44.0%)	0.476 (0.221–1.024)	0.058	0.076 (0.009–0.639)	0.018
TT	44 (29.3%)	24 (16.0%)	0.291 (0.115–0.737)	0.009	0.006 (0.000-0.258)	0.008
Dominant model (CT + TT vs. CC)			0.49 (0.32–0.75)	0.001		
Recessive model (TT vs. CC + CT)			0.46 (0.27–0.78)	0.004		

Allelic model	Control (*n* = 300)	MASLD (*n* = 300)	Crude OR (95% CI)	*P* value	Adjusted OR (95% CI)	*P* value
C	138 (46.0%)	186 (62.0%)	Reference		Reference	
T	162 (54.0%)	114 (38.0%)	0.522 (0.330–0.827)	0.006	0.137 (0.041–0.455)	0.001

MASLD versus clinically suspected MASH						
Codominant model	MASLD (*n* = 150)	MASH (*n* = 150)	Crude OR (95% CI)	*P* value	Adjusted OR (95% CI)	*P* value
CC	60 (40.0%)	26 (17.3%)	Reference		Reference	
CT	66 (44.0%)	68 (45.3%)	2.378 (1.060–5.334)	0.036	3.344 (1.482–7.545)	0.004
TT	24 (16.0%)	56 (37.3%)	5.385 (2.106–13.764)	< 0.001	16.875 (6.339–44.921)	< 0.001
Dominant model (CT + TT vs. CC)			3.18 (1.94–5.22)	< 0.001		
Recessive model (TT vs. CC + CT)			3.13 (1.78–5.50)	< 0.001		

Allelic model	MASLD (*n* = 300)	MASH (*n* = 300)	Crude OR (95% CI)	*P* value	Adjusted OR (95% CI)	*P* value
C	186 (62.0%)	120 (40%)	Reference			
T	114 (38.0%)	180 (60%)	2.447 (1.539–3.893)	< 0.001	4.353 (2.735–6.927)	< 0.001

Normal controls versus clinically suspected MASH						
Codominant model	Control (*n* = 150)	MASH (*n* = 150)	Crude OR (95% CI)	*P* value	Adjusted OR (95% CI)	*P* value
CC	32 (21.3%)	26 (17.3%)	Reference		Reference	
CT	74 (49.3%)	68 (45.3%)	1.131 (0.475–2.693)	0.781	-----	1.000
TT	44 (29.3%)	56 (37.3%)	1.566 (0.624–3.933)	0.339	-----	1.000
Allelic model	Control (*n* = 300)	MASH (*n* = 300)	Crude OR (95% CI)	P value	Adjusted OR (95% CI)	P value
C	138 (46.0%)	120 (40%)	Reference		Reference	
T	162 (54.0%)	180 (60%)	1.278 (0.808–2.020)	0.294	-----	0.999

Data are presented as numbers (percentage)s

**Table 3:** Comparison between GCKR *rs1260326* (C/T) genetic variants in MASLD and MASH patients regarding the laboratory data

Variable represented as mean ± SD	CC genotype	CT genotype	TT genotype	*P* value
Age (Years)	49.47 ± 9.58	48.34 ± 9.41	47.10 ± 7.28	0.486
Waist circumference	97.41 ± 6.48	94.34 ± 7.61	93.88 ± 6.60	0.157
Length (cm)	170.33 ± 6.92	171.34 ± 5.57	171.30 ± 3.82	0.614
Weight (kg)	87.81 ± 9.38	91.40 ± 9.38	90.53 ± 6.83	0.110
BMI (kg/m²)	30.27 ± 2.91	31.16 ± 3.13	30.88 ± 2.47	0.299
Hemoglobin (g/dl)	11.46 ± 1.50	11.24 ± 1.23	11.47 ± 1.64	0.628
TLC (×10⁹/L)	5.34 ± 1.60	4.90 ± 1.45	5.17 ± 1.62	0.326
Platelet count (×10³/µL)	167.74 ± 20.99	175.03 ± 31.70	181.70 ± 30.82	0.091
FBS (mg/dl)	94.56 ± 28.16	89.39 ± 22.47	89.18 ± 25.93	0.513
HbA1C (%)	5.41 ± 0.92	5.41 ± 0.81	5.31 ± 0.83	0.809
Insulin (µU/ml)	18.35 ± 8.10	19.28 ± 9.21	19.90 ± 8.60	0.716
HOMA-IR	4.60 ± 3.99	4.62 ± 4.24	5.50 ± 4.5	0.932
Total protein (g/dl)	8.88 ± 0.35	8.53 ± 0.33	6.68 ± 0.41	0.034
Albumin (g/dl)	4.51 ± 0.35	4.55 ± 0.41	4.54 ± 0.44	0.850
ALT (IU/L)	34.16 ± 9.35	38.88 ± 14.23	43.72 ± 17.07	0.009
AST (IU/L)	29.02 ± 8.62	32.25 ± 10.06	32.90 ± 12.02	0.167
Total blirubin (mg/dl)	1.02 ± 0.11	1.02 ± 0.09	1.04 ± 0.11	0.469
Direct blirubin (mg/dl)	0.56 ± 0.12	0.53 ± 0.11	0.53 ± 0.10	0.217
Uric acid (mg/dl)	5.01 ± 0.99	5.41 ± 1.22	5.41 ± 1.24	0.167
GGT (IU/L)	43.26 ± 10.97	46.13 ± 15.17	46.70 ± 18.18	0.515
Urea (mg/dl)	36.35 ± 7.64	37.00 ± 7.08	37.25 ± 6.18	0.829
Creatinine (mg/dl)	1.05 ± 0.17	1.05 ± 0.16	1.06 ± 0.15	0.889
Total cholesterol (mg/dl)	206.74 ± 45.52	199.18 ± 41.01	200.13 ± 40.88	0.637
HDL-C (mg/dl)	41.33 ± 9.42	39.84 ± 10.10	37.17 ± 9.83	0.154
LDL-C (mg/dl)	109.84 ± 12.64	106.94 ± 13.51	108.87 ± 14.61	0.525
Triglycerides (mg/dl)	149.02 ± 22.88	153.60 ± 24.12	152.98 ± 28.57	0.627

### Frequency distribution of FGF21 *rs838133* polymorphism and metabolic associations

**Fig. 4:**
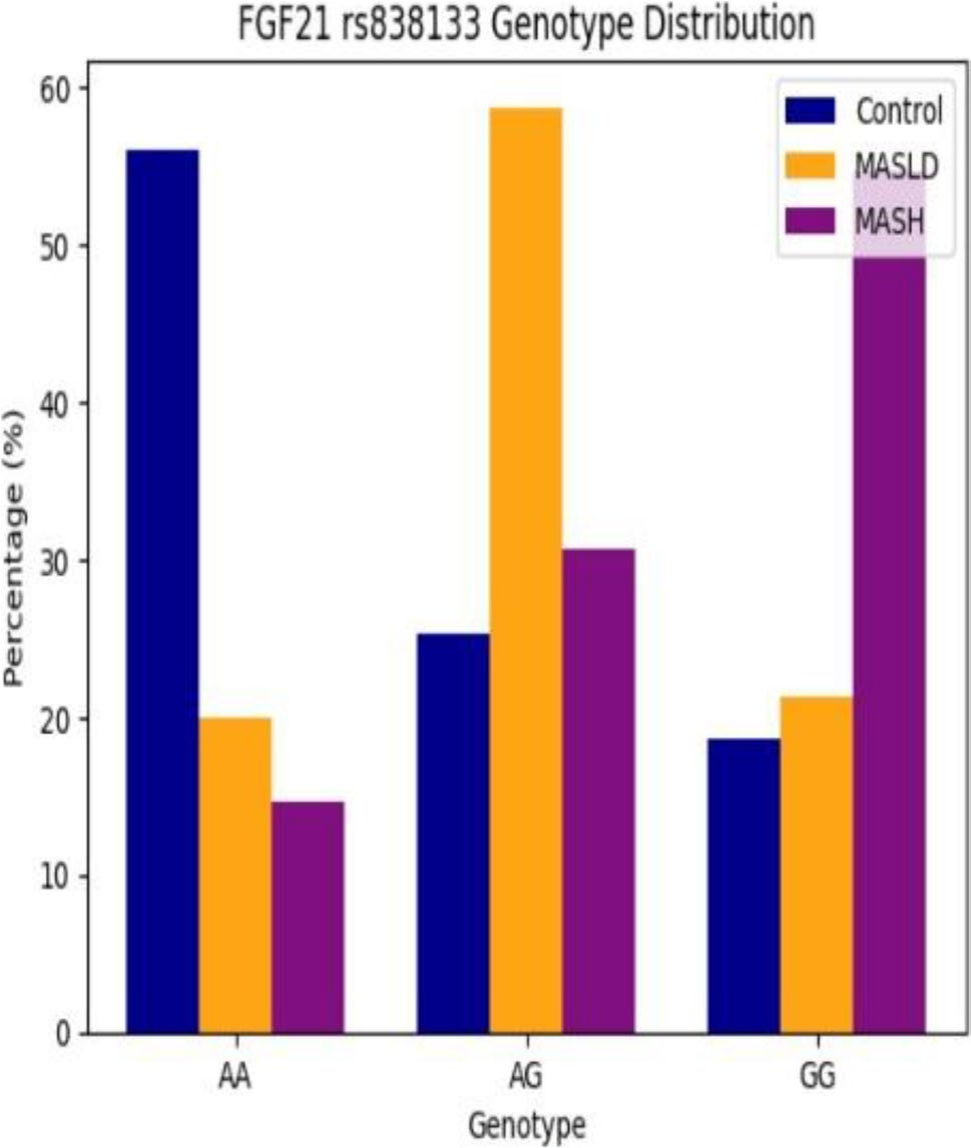
Genotypic distribution of *rs838133* SNP variants among the studied groups

**Fig. 5:**
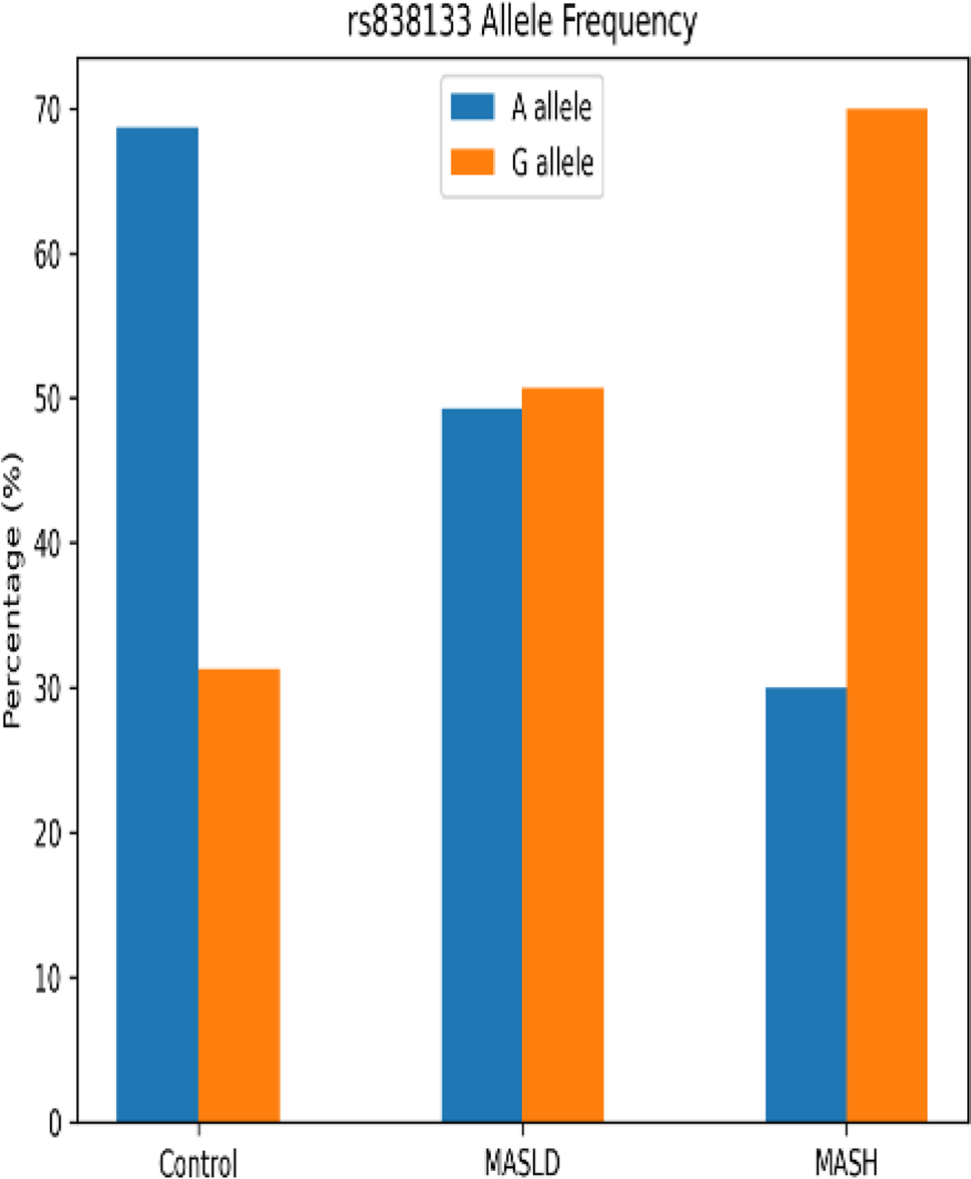
Allelic distribution of *rs838133* SNP variants among the studied groups

**Fig. 6:**
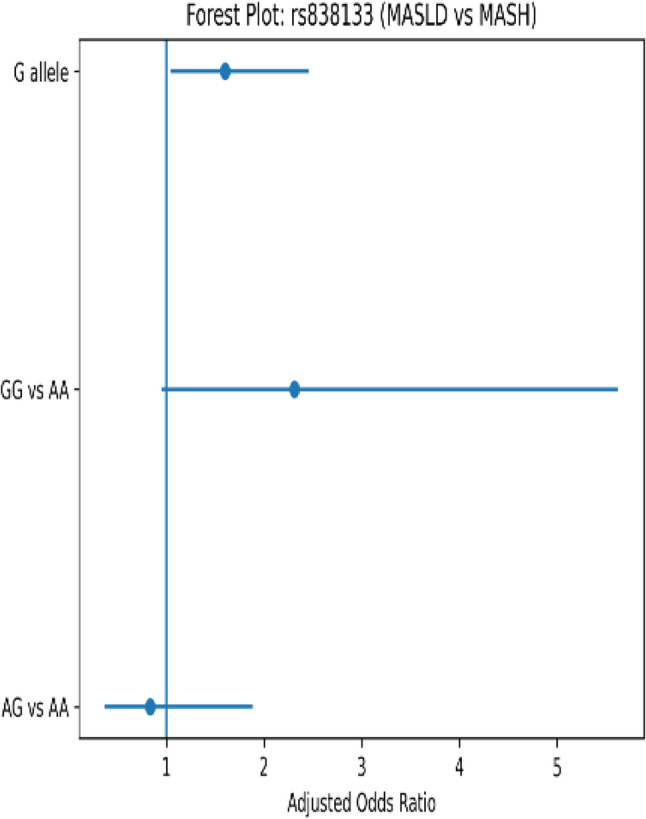
Forest plot of *rs838133* SNP variants in MASLD versus MASH

Genotypic analysis of the FGF21 *rs838133* variant showed a striking shift in allele distribution across the disease spectrum (Figs. 4, 5 and 6). The GG genotype and G allele frequencies were significantly higher in MASLD and MASH versus controls (P *<* 0.001). Post-adjustment, GG genotype showed reduced but non-significant association with MASH risk versus MASLD (AOR = 2.313, 95% CI: 0.950–5.628, *P* = 0.065), while G allele remained significant (AOR = 1.602, 95% CI: 1.043–2.459, *P* = 0.031) (Table 4). These adjusted associations highlight genetic risk for MASLD-to-MASH transition after confounder controls. While the AG genotype was significantly higher in MASLD patients compared with healthy controls. Post-adjustment, the AG genotype carriers conferring 18.622-folds higher risk for MASLD versus healthy controls (95% CI: 1.619-214.169, *P* = 0.019) (Table 4). As well genotype frequencies in the control group were in Hardy–Weinberg equilibrium (*p* = 0.74).

In patients with clinically suspected MASH, the GG genotype was independently associated with higher LDL-cholesterol levels (*P* = 0.035) and greater insulin resistance as reflected by increased HOMA-IR values (*P* = 0.004); suggesting metabolic contributions to progression (Table 5). Among patients with MASLD, ultrasonographic assessment revealed a significantly higher prevalence of hepatic steatosis in GG carriers compared with those harboring the AA or AG genotypes (*p* = 0.049), supporting a role for this variant in early disease susceptibility.

**Table 4:** Genotype and Allele distribution of *rs838133* SNP genotypic variants and their odd’s ratios among the studied groups

Genotypes	Control (*n* = 150)		MASLD (*n* = 150)		MASH (*n* = 150)	*P* value
AA	84 (56%)		30 (20%)		22 (14.7%)	*< 0.001*
AG	38 (25.3%)		88 (58.7%)		46 (30.7%)	
GG	28 (18.7%)		32 (21.3%)		82 (54.7%)	
Alleles	Control (*n* = 300)		MASLD (*n* = 300)		MASH (*n* = 300)	*P value*
A allele	206 (68.7%)		148 (49.3%)		90 (30%)	*< 0.001*
G allele	94 (31.3%)		152 (50.7%)		210 (70%)	

Normal controls versus MASLD						
Codominant model	Control (*n* = 150)	MASLD (*n* = 150)	Crude OR (95% CI)	*P* value	Adjusted OR (95% CI)	*P* value
AA	84 (56%)	30 (20%)	Reference		Reference	
AG	38 (25.3%)	88 (58.7%)	6.484 (3.688–11.402)	< 0.001	18.622 (1.619-214.169)	0.019
GG	28 (18.7%)	32 (21.3%)	3.200 (1.660–6.170)	0.001	2.186 (0.251–22.316)	0.508
Dominant model (AG + GG vs. AA)			5.00 (3.00-8.33)	< 0.001		
Recessive model (GG vs. AA + AG)			0.91 (0.5–1.66)	0.75		

Allelic model	Control (*n* = 300)	MASLD (*n* = 300)	Crude OR (95% CI)	*P* value	Adjusted OR (95% CI)	*P* value
A allele	206 (68.7%)	148 (49.3%)	Reference		Reference	
G allele	94 (31.3%)	152 (50.7%)	2.251 (1.406–3.603)	< 0.001	1.610 (0.562–4.614)	0.376

MASLD versus clinically suspected MASH						
Codominant model	MASH (*n* = 150)	MASLD (*n* = 150)	Crude OR (95% CI)	*P* value	Adjusted OR (95% CI)	*P* value
AA	22 (14.7%)	30 (20%)	Reference		Reference	
AG	46 (30.7%)	88 (58.7%)	0.713 (0.282–1.802)	0.474	0.836 (0.371–1.883)	0.666
GG	82 (54.7%)	32 (21.3%)	3.494 (1.326–9.209)	0.011	2.313 (0.950–5.628)	0.065
Dominant model (AG + GG vs. AA)			3.77 (2.00-6.46)	< 0.001		
Recessive model (GG vs. AA + AG)			4.74 (2.80-8.00)	< 0.001		

Allelic model	MASH (*n* = 300)	MASLD (*n* = 300)	Crude OR (95% CI)	*P* value	Adjusted OR (95% CI)	*P* value
A allele	90 (30%)	148 (49.3%)	Reference		Reference	
G allele	210 (70%)	152 (50.7%)	2.272 (1.415–3.649)	< 0.001	1.602 (1.043–2.459)	0.031

Normal controls versus clinically suspected MASH						
Codominant model	Control (*n* = 150)	MASH (*n* = 150)	Crude OR (95% CI)	*P* value	Adjusted OR (95% CI)	*P* value
AA	84 (56%)	22 (14.7%)	Reference		Reference	
AG	38 (25.3%)	46 (30.7%)	4.622 (1.879–11.368)	< 0.001	--------	1.000
GG	28 (18.7%)	82 (54.7%)	11.182 (4.549–27.484)	< 0.001	--------	1.000

Allelic model	Control (*n* = 300)	MASH (*n* = 300)	Crude OR (95% CI)	*P* value	Adjusted OR (95% CI)	*P* value
A allele	206 (68.7%)	90 (30%)	Reference		Reference	
G allele	94 (31.3%)	210 (70%)	5.113 (3.130–8.354)	< 0.001	--------	1.000

Data are presented as numbers (percentage)

**Table 5:** Comparison between FGF21 *rs838133* (A/G) genetic variants in MASLD and MASH patients regarding the laboratory data

Variable represented as mean ± SD	AA (Mean ± SD)	AG (Mean ± SD)	GG (Mean ± SD)	*P* value
Age (Years)	46.09 ± 10.31	47.58 ± 8.52	49.99 ± 8.26	0.089
Waist circumference	91.79 ± 8.48	94.60 ± 7.11	93.51 ± 6.19	0.209
Length (cm)	170.62 ± 9.54	171.69 ± 9.10	170.72 ± 5.64	0.626
Weight (kg)	87.67 ± 8.94	91.73 ± 9.10	90.28 ± 7.95	0.114
BMI (kg/m²)	30.08 ± 3.45	31.13 ± 2.96	30.99 ± 2.64	0.225
Hemoglobin (g/dl)	12.90 ± 1.87	13.22 ± 1.49	13.26 ± 1.22	0.444
TLC (×10⁹/L)	4.64 ± 1.25	5.41 ± 1.64	5.11 ± 1.56	0.086
Platelet count (×10³/µL)	173.76 ± 28.88	178.41 ± 28.74	173.37 ± 29.75	0.756
FBS (mg/dl)	97.44 ± 10.33	87.10 ± 10.26	90.12 ± 12.55	0.114
HbA1C (%)	5.55 ± 1.14	5.27 ± 0.77	5.39 ± 0.71	0.341
Insulin (µU/ml)	19.47 ± 11.72	20.02 ± 7.73	18.44 ± 7.60	0.618
HOMA-IR	4.46 ± 3.26	4.66 ± 3.62	5.68 ± 4.75	0.004
Total protein (g/dl)	8.02 ± 0.32	8.37 ± 0.37	8.76 ± 0.39	0.447
Albumin (g/dl)	4.51 ± 0.38	4.57 ± 0.35	4.57 ± 0.36	0.691
ALT (IU/L)	35.62 ± 13.58	39.96 ± 16.10	39.62 ± 13.14	0.330
AST (IU/L)	21.49 ± 10.52	26.14 ± 11.20	32.91 ± 9.57	0.118
Total blirubin (mg/dl)	1.03 ± 0.11	1.02 ± 0.10	1.01 ± 0.10	0.815
Direct blirubin (mg/dl)	0.52 ± 0.36	0.49 ± 0.31	0.53 ± 0.12	0.826
Uric acid (mg/dl)	5.16 ± 1.03	5.47 ± 1.31	5.23 ± 0.89	0.314
GGT (IU/L)	44.24 ± 13.85	45.94 ± 16.62	45.74 ± 14.47	0.683
Urea (mg/dl)	34.30 ± 8.62	35.43 ± 6.35	35.16 ± 8.02	0.514
Creatinine (mg/dl)	1.02 ± 0.19	1.08 ± 0.14	1.07 ± 0.16	0.383
Total cholesterol (mg/dl)	195.97 ± 39.85	198.35 ± 41.27	208.75 ± 48.02	0.133
HDL-C (mg/dl)	40.68 ± 8.70	40.49 ± 9.76	37.44 ± 10.71	0.199
LDL-C (mg/dl)	104.88 ± 8.81	106.33 ± 11.91	111.37 ± 9.35	0.035
Triglycerides (mg/dl)	149.03 ± 24.79	153.68 ± 22.81	155.40 ± 26.60	0.370

### Interaction between GCKR and FGF21 genetic variants

Analysis of the genotypic distribution between the FGF21 rs8387133 (A/G) and GCKR rs1260326 (C/T) polymorphisms revealed a statistically significant association between the two loci (*P* = 0.036, Table 6). Notably, the GCKR CC genotype was most prevalent in patients with the FGF21 GG genotype (21.2%), while the GCKR TT genotype was most prevalent in those with the FGF21 AG genotype (56.7%). In contrast, individuals with the FGF21 AA genotype exhibited a higher frequency of the GCKR CT genotype (50%). These findings suggest that the co-inheritance of specific alleles is not random and may amplify the genetic predisposition associated with these loci.

**Table 6:** Association between FGF-21 *rs838133* (A/G) and GCKR rs1260326 (C/T) genotypes

Variable represented as frequency (%)	FGF-21 (AA) genotype	FGF-21 (AG) genotype	FGF-21 (GG) genotype	*P* value
GCKR (CC) genotype	8 (33%)	4 (6.7%)	14 (21.2%)	0.036
GCKR (CT) genotype	12 (50%)	22 (36.6%)	34 (51.5%)	
GCKR (TT) genotype	4 (17%)	34 (56.7%)	18 (27.3%)	

Data are presented as numbers (percentage)

### Circulating FGF21 levels across disease stages

**Fig. 7:**
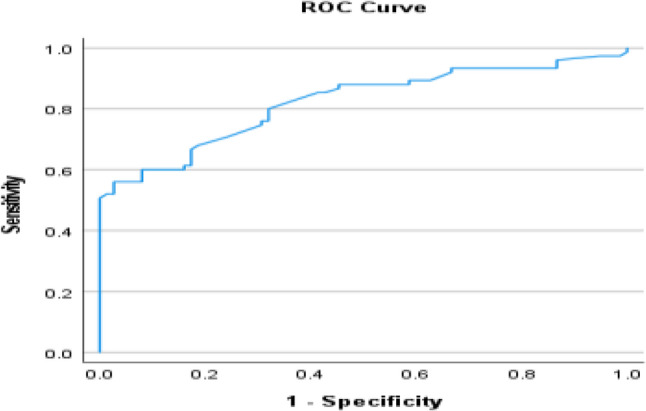
ROC for FGF-21 best cutoff to diagnose MASLD from control

**Fig. 8:**
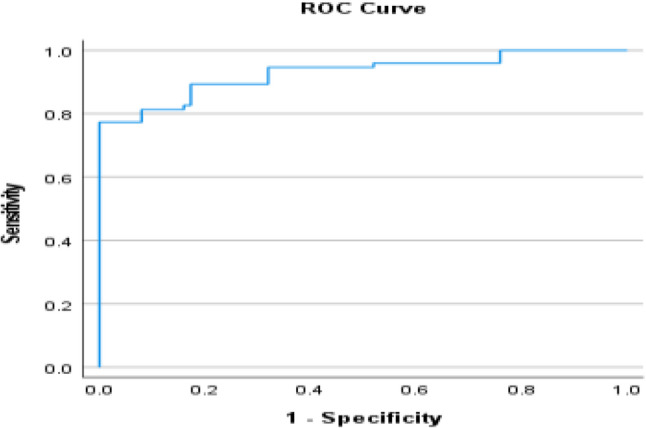
ROC for FGF-21 best cutoff to diagnose MASH from control

**Fig. 9:**
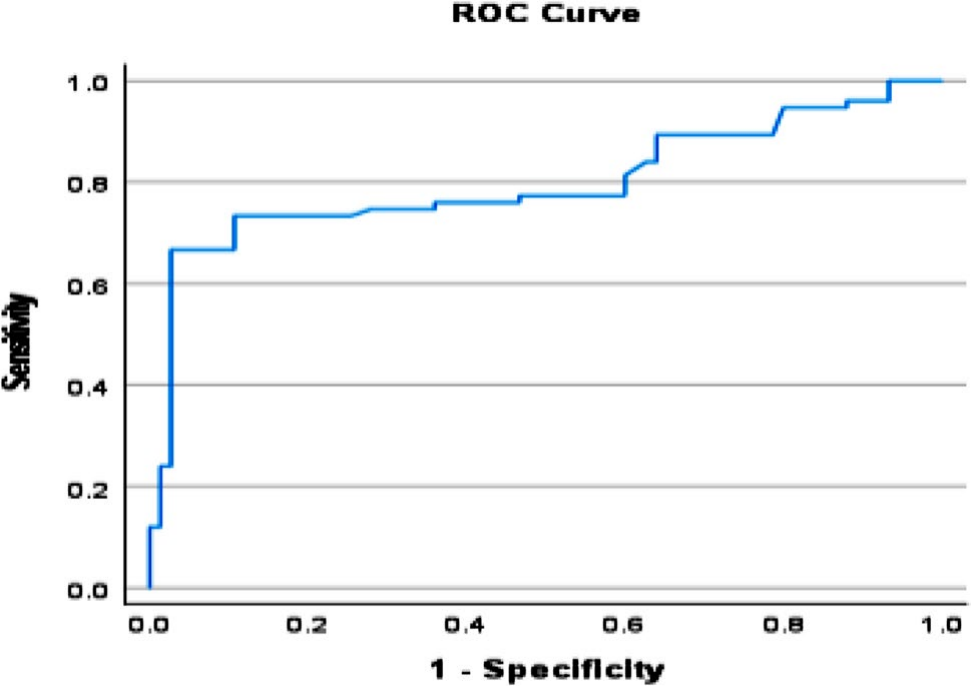
ROC for FGF-21 best cutoff to diagnose MASH from MASLD

Serum FGF21 concentrations increased progressively across the three study groups, with the lowest levels observed in healthy controls, intermediate levels in MASLD patients, and the highest concentrations in those with clinically suspected MASH (*p* < 0.001). Median FGF21 levels rose nearly threefold from controls to MASLD and more than sixfold in patients with clinically suspected MASH, reflecting a strong association between circulating FGF21 and disease severity (Table 7). Receiver operating characteristic (ROC) analysis demonstrated that serum FGF21 may be associated with MALSD progression. The biomarker showed good discriminatory performance in differentiating MASLD from controls (AUC = 0.821) and excellent accuracy in identifying clinically suspected MASH among healthy individuals (AUC = 0.929). Although sensitivity was moderate, specificity consistently exceeded 95%, underscoring the potential utility of FGF21 as a confirmatory marker for advanced disease rather than a screening tool (Figs. 7, 8 and 9).

**Table 7:** Median and interquartile range (IQR) of circulating FGF-21 levels in pg/mL among the three studied groups

Median and interquartile range (IQR) of circulating FGF-21 levels in pg/mL					
Controls (*n* = 150)		MASLD (*n* = 150)	MASH (*n* = 150)		*P* value
37.0 (21.0–64.0)		107.0 (61.0-156.0)	227.0 (121.0-369.0)		*< 0.001*
Controls versus MASLD	AUC	95% CI	Cut off	Sensitivity %	Specificity %
	0.821	0.753–0.890	98.5	56	97.3
Controls versus MASH	AUC	95% CI	Cut off	Sensitivity %	Specificity %
	0.929	0.886–0.971	106	77.3	100
MASLD versus MASH	AUC	95% CI	Cut off	Sensitivity %	Specificity %
	0.799	0.724–0.875	189.5	66.7	96

### Correlation analysis of serum FGF21 with metabolic and hepatic parameters

Distinct correlation patterns were observed across study groups. In healthy controls, serum FGF21 levels exhibited a weak but significant positive correlation with γ-glutamyl transferase (*r* = 0.279, *p* = 0.015), suggesting that even subtle variations in hepatic metabolic activity may influence FGF21 secretion under physiological conditions. No other significant associations were detected in this group. Among patients with MASLD, FGF21 concentrations showed no meaningful correlations with most metabolic or biochemical indices, with the exception of a weak inverse relationship with hemoglobin levels (*r* = -0.227, *p* = 0.050). In contrast, a different profile emerged in patients with clinically suspected MASH, in whom elevated FGF21 levels were positively correlated with insulin resistance, as reflected by HOMA-IR values (*r* = 0.244, *p* = 0.035). Although trends toward positive associations with fasting insulin and HbA1c were noted, these did not reach statistical significance. Collectively, these findings suggest that the clinical relevance of circulating FGF21 becomes more pronounced with increasing disease severity.

Moreover, the combined contribution of additional lipid-related genetic variants (MARC1 *rs2642438* A > T and TM6SF2 *rs58542926* C > T) concurrently reported in with our previously reported genetic associations in the same population, highlights the polygenic framework underlying MASH susceptibility, in which multiple modest-effect alleles collectively shape individual trajectories toward advanced liver disease.

Multivariable logistic regression analysis identified serum FGF21 levels as an independent predictor of MASH (B = 0.011, *p* = 0.001, OR = 1.011, 95% CI: 1.005–1.018). Similarly, carriers of the GCKR rs1260326 (TT + CT) genotype demonstrated an increased risk of MASH (B = 1.690, *p* = 0.039, OR = 5.420, 95% CI: 1.085–27.062). Similarly, the TM6SF2 rs58542926 variant showed a strong association with MASH (CT: OR = 9.956, 95% CI: 4.373–22.665; TT: OR = 18.667, 95% CI: 5.537–62.936; both *p* < 0.001). Conversely, the MARC1 rs2642438 (TT) genotype exhibited a protective effect against MASH (B = − 2.289, *p* = 0.013, OR = 0.101, 95% CI: 0.017–0.614). As well, ALT levels were positively associated with MASH risk B = 0.530, *p* < 0.001, OR = 1.699, 95% CI: 1.304–2.213), whereas cholesterol levels showed a modest inverse association (B = − 0.030, *p* = 0.002, OR = 0.971, 95% CI: 0.952–0.989).

The final predictive regression equation was expressed as:

$$\begin{aligned} \mathrm{Logit}\;\mathrm{(P)} =\: &0.011\times\mathrm{FGF21} + 1.690\times\mathrm{GCKR}\;\mathrm{rs1260326}\;\mathrm{(TT+CT)} \\&- 2.289\times\mathrm{MARC1}\;\text {rs2642438}\;\mathrm{(TT)} \\&- 2.127\times\mathrm{TM6SF2}\;\mathrm{rs58542926}\;\mathrm{(TT+CT)} \\&+ 0.530\times\mathrm{ALT} - 0.030\times\mathrm{Chol} - 15.071 \end{aligned}$$

## Discussion

The present study provides integrative evidence that disease progression is shaped by the convergence of metabolic burden, inherited genetic susceptibility, and dysregulated hepatokine signaling. By combining genetic variants in GCKR and FGF21 with circulating FGF21 concentrations, our findings move beyond single-marker approaches and offer a more refined framework for risk stratification in MASLD.

The rs1260326 (P446L) variant in GCKR is functionally characterized by reduced inhibitory control over hepatic glucokinase, leading to enhanced glycolytic flux and increased de novo lipogenesis. This mechanistic shift provides a plausible explanation for the observed association between the TT genotype and disease progression from MASLD to MASH as we found that TT genotype carriers had a 16.875-fold increased risk of MASH progression versus MASLD, with T allele conferring 4.353-fold risk. Interestingly, the observed pattern of the GCKR rs1260326 variant suggests a potential stage-dependent role. While the TT genotype did not appear to increase susceptibility to MASLD compared to controls, it was relatively enriched in patients with clinically suspected MASH. This apparent duality highlights a context-specific role of GCKR polymorphisms in disease biology, exerting divergent effects on disease initiation rather than initiation. Nevertheless, this interpretation remains speculative and requires validation in larger, longitudinal studies. Our findings align with previous reports, including those by *Samarasinghe et al.*, who demonstrated that the T allele was associated with higher grades of hepatic steatosis in Indian patients, supporting the concept that this variant may promote inflammatory progression once steatosis is established [20]. Collectively, these observations suggest that GCKR may function not merely as a susceptibility locus, but as a metabolic switch influencing the trajectory of MASLD across disease stages. The biochemical signature observed in TT carriers characterized by higher aminotransferase levels and reduced total protein concentrations, suggests that this variant contributes not only to steatosis but also to impaired hepatocellular function as disease advances [21]. These findings are consistent with previous reports by *Nisar et al.* [22], linking GCKR rs1260326 to hepatic fat accumulation and more severe metabolic phenotypes, reinforcing its role as a genetic modifier of disease severity rather than mere disease presence.

In parallel, our data highlight a substantial contribution of FGF21 rs838133 genetic variability to MASLD susceptibility and progression. Carriers of the GG genotype exhibited a markedly increased risk of both MASLD and MASH, as G allele carriers had a 1.6-fold increased risk of MASH progression versus MASLD, and AG genotype carriers conferring 18.622-folds higher risk for MASLD versus healthy controls. This is accompanied by greater insulin resistance and adverse lipid profiles, particularly elevated LDL-cholesterol levels. While FGF21 is widely recognized as locus may compromise the effectiveness of this adaptive response. The higher prevalence of hepatic steatosis among GG carriers within the MASLD group further supports a role for this variant early in disease development, potentially predisposing individuals to metabolic dysregulation before overt inflammation or fibrosis becomes evident. These observations align with emerging genetic and experimental data by *Ramne et al.* [23], indicating that impaired FGF21 signaling may contribute to metabolic inflexibility and hepatic lipid overload.

A novel aspect of the present study is the demonstration of a gene–gene interaction between GCKR and FGF21 variants. The non-random co-distribution of high-risk genotypes suggests a synergistic effect that may amplify metabolic disturbances beyond the impact of either variant alone. From a mechanistic standpoint, enhanced hepatic glycolytic flux driven by GCKR variation could increase lipid synthesis and metabolic stress, thereby stimulating FGF21 secretion. If this compensatory pathway is genetically compromised, the imbalance between lipid accumulation and adaptive capacity may accelerate hepatocellular injury and fibrogenesis; such findings support the integrated genetic model identified by *Singh et al.* [24], . This multilocus interaction provides a plausible explanation for the marked interindividual variability observed in MASLD progression and supports polygenic models of disease susceptibility.

Beyond genetic determinants, circulating FGF21 concentrations emerged as a robust biochemical marker of disease severity. Serum FGF21 levels increased progressively from healthy controls to MASLD and MASH, reflecting escalating hepatic and systemic metabolic stress. Importantly, receiver operating characteristic analyses demonstrated high specificity for advanced disease, indicating that elevated FGF21 levels may be particularly useful for identifying clinically suspected patients with MASH rather than for population-wide screening and representing a meaningful clinical improvement beyond ALT levels. Our findings demonstrated a pattern which aligns with *Filimidou et al.*, in which serum FGF21 exhibits strong discriminatory capacity for identify patients with fatty liver disease compared with healthy subjects (AUC ≈ 0.83) [25]. *Gallego-Durán et al.*, further demonstrated that both hepatic and circulating FGF21 levels were significantly elevated in clinically suspected MASH patients, in Huh7.5 cells exposed to free fatty acids, and in CDA-HFD animal model, corroborating our observations [10]. In the present study, elevated circulating FGF21 levels were associated with increasing disease severity. Rather than interpreting this finding as definitive evidence of FGF21 resistance, we consider it more appropriate to view FGF21 as a marker of metabolic stress response. A compensatory upregulation mechanism in response to hepatic and systemic metabolic disturbances is plausible. However, given the cross-sectional nature of the study, causal interpretation remains limited.

Our findings support a polygenic framework underlying MASH susceptibility, in which multiple lipid-related variants exert cumulative and potentially interacting effects on disease trajectory. While GCKR and TM6SF2 variants were associated with increased risk of MASH, MARC1 appeared to confer a protective effect, highlighting the complex and sometimes opposing biological influences shaping disease progression. Importantly, the integration of genetic variants with circulating hepatokines such as FGF21 significantly enhanced risk discrimination. This suggests that MASH progression is not driven by isolated genetic effects, but rather by a coordinated metabolic–genetic network. The derived regression model further underscores the additive contribution of these variants within a unified predictive framework. Such an approach aligns with emerging precision-based strategies in MASLD, where combined genetic and metabolic profiling may allow earlier identification of high-risk individuals.

In addition to fibroblast growth factor 21, several circulating biomarkers involved in metabolic and inflammatory pathways have been explored. Among these, Lipocalin-2 is a secreted glycoprotein that has been reported to be overexpressed in various cancers and has been proposed as a potential serum biomarker associated with metabolic dysregulation and inflammatory responses, suggesting a possible association with disease pathogenesis. Previous experimental studies have investigated the biological effects of targeting LCN2 in inflammatory breast cancer (IBC) cells using small interfering RNAs (siRNAs) and small-molecule inhibitors. Silencing of LCN2 in IBC cells significantly reduced cell proliferation, viability, migration, and invasion. Moreover, LCN2 suppression promoted apoptosis and induced cell-cycle arrest at the G0/G1–S phase transition [26].

This study has several important limitations. First, the diagnosis of clinically suspected MASH was based on non-invasive criteria rather than histological confirmation. Although this approach aligns with current clinical practice, it may have resulted in misclassification, particularly in distinguishing early steatohepatitis from simple steatosis. This limitation could have influenced the observed genotype–phenotype associations and should be considered when interpreting the findings. Second, the case–control design of the study, causal relationships cannot be definitively established. Although genetic variants are temporally stable, the observational nature of the study limits inference regarding disease progression. Therefore, the observed associations should be interpreted as hypothesis-generating rather than conclusive evidence of mechanistic relationships. Third, despite adjustment for major metabolic confounders, residual confounding cannot be excluded as medication use was not systematically recorded in this study. Given that glucose-lowering, lipid-lowering, antihypertensive therapies, as well as weight-loss interventions, may influence metabolic parameters and circulating FGF21 levels. Additionally, the relatively modest sample size may have contributed to variability in effect estimates, particularly for subgroup analyses. Finally, external validation in independent studies, ideally with biopsy-confirmed MASH and longitudinal follow-up, is required to confirm the robustness and clinical applicability of our findings. Nevertheless, the strength of this study lies in its integrative design, combining genetic, biochemical, and clinical data to provide a more comprehensive characterization of disease heterogeneity.

## Conclusion

Progression from MASLD to MASH appears to be driven by the interplay between metabolic stress, inherited susceptibility, and adaptive hepatokine responses. Our findings suggest that GCKR rs1260326 and FGF21 rs838133 are associated with disease severity in MASLD and clinically suspected MASH, and may may contribute to a metabolic risk signature reflecting disease progression. Circulating FGF21 levels further reflect this interaction, serving as a marker of disease burden and a potential tool for clinical risk assessment. However, given the limitations of non-invasive classification, these results should be interpreted with caution and warrant validation in larger, biopsy-confirmed and longitudinal cohorts.

## Data Availability

The data is available upon reasonable request from the corresponding author.

## Abbreviations

MASLD: Metabolic dysfunction–associated steatotic liver disease
MASH: Metabolic dysfunction–associated steatohepatitis
GCKR: Glucokinase regulatory protein
FGF21: Fibroblast growth factor 21
ALT: Alanine aminotransferase
AST: Aspartate aminotransaminase
ALP: Alkaline phosphatase
GGT: ᵞ-glutamyl transferase
HDL-C: High‐density lipoprotein cholesterol
LDL-C: Low‐density lipoprotein cholesterol
HOMA-IR: Homeostatic model assessment of insulin resistance
BMI: Body mass index
IRB: Institutional Review Board
ELISA: Enzyme-linked immunosorbent assay
q-PCR: quantitative polymerase chain reaction OR
OR: Crude odds ratio
AOR: Adjusted odds ratio
AUC: Area under the curve
ROC: Receiver operating characteristic
